# DNA polymerase ζ in DNA replication and repair

**DOI:** 10.1093/nar/gkz705

**Published:** 2019-08-14

**Authors:** Sara K Martin, Richard D Wood

**Affiliations:** Department of Epigenetics & Molecular Carcinogenesis, The University of Texas MD Anderson Cancer Center, Smithville, TX, USA and The University of Texas MD Anderson Cancer Center UT Health Graduate School of Biomedical Sciences

## Abstract

Here, we survey the diverse functions of DNA polymerase ζ (pol ζ) in eukaryotes. In mammalian cells, REV3L (3130 residues) is the largest catalytic subunit of the DNA polymerases. The orthologous subunit in yeast is Rev3p. Pol ζ also includes REV7 subunits (encoded by *Rev7* in yeast and *MAD2L2* in mammalian cells) and two subunits shared with the replicative DNA polymerase, pol δ. Pol ζ is used in response to circumstances that stall DNA replication forks in both yeast and mammalian cells. The best-examined situation is translesion synthesis at sites of covalent DNA lesions such as UV radiation-induced photoproducts. We also highlight recent evidence that uncovers various roles of pol ζ that extend beyond translesion synthesis. For instance, pol ζ is also employed when the replisome operates sub-optimally or at difficult-to-replicate DNA sequences. Pol ζ also participates in repair by microhomology mediated break-induced replication. A *rev3* deletion is tolerated in yeast but *Rev3l* disruption results in embryonic lethality in mice. Inactivation of mammalian *Rev3l* results in genomic instability and invokes cell death and senescence programs. Targeting of pol ζ function may be a useful strategy in cancer therapy, although chromosomal instability associated with pol ζ deficiency must be considered.

## INTRODUCTION

**Figure 1.**
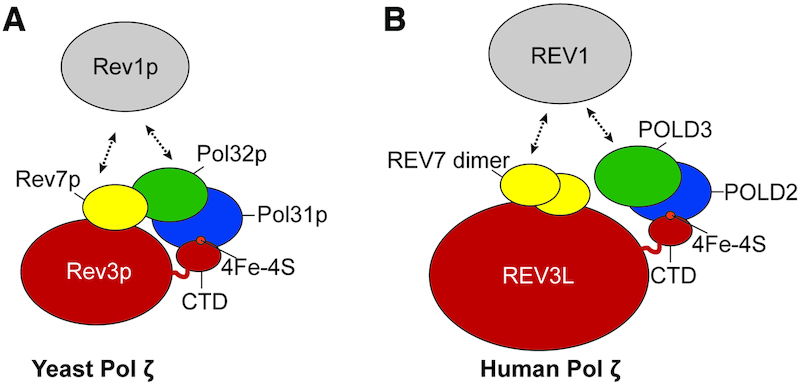
Pol ζ is a multi-subunit polymerase that interacts with the TLS master regulator, REV1. (**A**) Schematic of pol ζ from the budding yeast *Saccharomyces cerevisiae*. The catalytic subunit Rev3p binds to the accessory subunit Rev7p. The precise binding sites and dimeric state of Rev7p are unknown. Pol δ accessory subunits, Pol31p-Pol32p, bind to the C-terminal domain (CTD). Pol31p interaction is coordinated by the iron-sulfur cluster (4Fe-4S) in Rev3p. Rev7p and Pol32p interact directly. Rev7p and Pol32p mediate the interaction with Rev1p. (**B**) Schematic of human pol ζ. A dimer of REV7 can bind to the catalytic subunit, REV3L. POLD2 binds REV3L at the CTD, coordinated by the iron-sulfur (4Fe-4S) cluster. POLD3 binds to POLD2. It is unknown whether REV7 and POLD3 directly interact as Rev7p and Pol32p do in yeast. REV1 can bind human pol ζ through interactions with REV7 and POLD3.

DNA polymerase ζ (pol ζ) is universally present in eukaryotes including fungi, plants, and animals. Genetic studies of the budding yeast *Saccharomyces cerevisiae* have long established that pol ζ is a biologically fundamental enzyme, necessary for mediating most damage-induced mutagenesis. In this summary, we highlight newer discoveries of multiple roles for pol ζ. Pol ζ is a multi-subunit enzyme (Figure 1). The catalytic subunit is called Rev3p in budding yeast *S. cerevisiae*, and has 1504 amino acid residues. In mammalian cells, the orthologous protein REV3L is twice the size (3130 amino acids in human cells). Although the mammalian and yeast enzymes have some basic biochemical similarities, there are also significant functional differences, as summarized below.

### Pol ζ structure and composition

**Figure 2.**
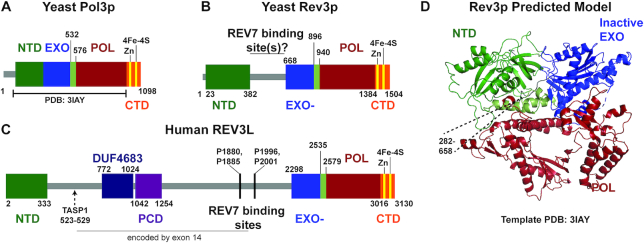
Rev3p/REV3L is a B-family DNA polymerase. Schematic of the catalytic subunits of (**A**) pol δ (Pol3p), (**B**) yeast pol ζ (Rev3p) and (**C**) human pol ζ (REV3L). All contain a conserved N-terminal domain (NTD, dark and light green), an exonuclease domain (EXO, blue), a B-family polymerase domain (POL, red), and a C-terminal domain (CTD, orange) with two metal binding sites: one that binds zinc (CysA) and one that binds an iron-sulfur cluster (4Fe-4S, CysB). The NTD is annotated by Interpro as domain IPR006133. The EXO domain is active in pol δ but inactive in pol ζ (EXO-). Rev7p binding likely occurs somewhere in between the NTD and the EXO- in Rev3p. In human REV3L, exon 14 encodes a large insertion that contains a positively charged domain (PCD, purple) and a domain of unknown function (DUF4683). Two REV7 binding sites (13) are well-conserved in mammals. (**D**) We generated a predicted model of Rev3p using PHYRE2 (125), with the known structure of Pol3p (3) as a template (PDB: 3IAY) given that Rev3p shares the highest sequence identity with pol δ. This shows the predicted folding of the NTD with the catalytic core of REV3L. The insert between the NTD and EXO- domains (282-658) was not included in the predicted model.

Rev3p/REV3L is a member of the ‘B-family’ of DNA polymerases. The other B-family DNA polymerases in eukaryotes (Pols α, δ and ε, Figure 2A) are components of the core DNA replication apparatus. The C-terminal portion of Rev3p/REV3L encompasses most of the conserved DNA polymerase domain (Figure 2B, C). A conserved N-terminal domain is predicted to form part of the overall polymerase fold (1,2) (Figure 2D).

Structures of the homologous regions of other B-family DNA polymerases show that the N-terminal domain caps the exonuclease domain, and contains residues that make direct contacts to DNA (2–5). In the other B-family polymerases, the DNA sequence encoding the N-terminal domain is directly adjacent to that encoding the exonuclease domain (Figure 2A). However, for Rev3p/REV3L and its orthologs, a large insertion separates the N-terminal domain from an inactive exonuclease domain (Figure 2B, C). In yeast pol δ, a ∼45 amino acid region immediately downstream of the exonuclease domain (Figure 2A, light green) also folds with the N-terminal domain (3). This region is conserved and structural modeling predicts that it folds similarly with the N-terminal domain in Rev3p (Figure 2B–D).

A single exon encodes the large central insertion that interrupts the domains of the catalytic core, and its variation across species results in large differences in the protein size. In mammalian *REV3L* genes this exon is relatively gargantuan (1386 amino acids), resulting in a protein double the size of yeast Rev3p (6) (Figure 2A).

While this single exon insert contains many stretches of predicted disorder, it is also home to a conserved positively charged domain that is necessary for efficient polymerase activity of the recombinant protein (7). A ∼250 aa region of the large insert of REV3L is highly homologous to the *NEXMIF (KIAA2022)* gene (6). This is annotated as Pfam domain DUF4683. The role this domain plays in REV3L or NEXMIF function is uncertain.

Like the other B-family polymerases, pol ζ contains multiple accessory subunits, including two subunits of pol δ: Pol31p/POLD2 and Pol32p/POLD3. The C-terminal domain of Rev3p/REV3L directly binds Pol31p/POLD2 (7–10) (Figure 1). The C-terminal domain contains two cysteine clusters: one that binds zinc (CysA) and one that binds an iron-sulfur [4Fe–4S] cluster (CysB) (8) (Figure 2A–C). CysB is essential for the binding of Pol31p/POLD2 to Rev3p/REV3L and pol ζ function (7,9,10). Pol32p/POLD3 binds indirectly through its interaction with Pol31p/POLD2. Pol ζ includes a third accessory subunit, Rev7p/REV7 (mammalian gene name *MAD2L2)* (11).

In mammalian REV3L there are two adjacent binding sites for REV7 with a consensus ϕϕxPxxxxPSR (where ϕ represents an aliphatic amino acid residue) (12,13). One of these mammalian binding sites resides in the large exon (Figure 2C). Crystal structures of REV7 bound to the two corresponding REV3L peptides have been obtained (14,15). Dimerization of REV7 appears to contribute to pol ζ biological function in mammalian cells (15,16). The exact interaction site or sites have yet to be discovered in yeast, and it is unknown whether Rev7p forms a dimer in yeast pol ζ. Rev7p greatly enhances pol ζ activity *in vitro* through an unknown mechanism (17). Similarly, Pol31p/POLD2 and Pol32p/POLD3 stimulate the activity of Rev3p/REV3L-Rev7p/REV7 (7,10).

A low resolution structure (23 Å) of yeast pol ζ obtained by negative stain electron microscopy suggests that all three regulator subunits form a hub, with Rev7p making direct contact to Pol32p (2) (Figure 1). Whether POLD3 interacts with REV7 in mammals has not been reported. The function of this hub in pol ζ remains largely unexplored. One important role is that it may serve to regulate recruitment of Rev1p/REV1, a major regulator of translesion DNA synthesis (TLS) (Figure 1). As will be discussed later, proper pol ζ function requires interaction with Rev1p/REV1. Rev1p/REV1 binds to pol ζ through direct interactions with both Rev7p/REV7 and Pol32p/POLD3 (12,18–23).

### *REV3L* expression and isoforms

*REV3L* is ubiquitously expressed in many tissues to yield a low level of protein. There are two main isoforms of the human and mouse mRNA transcripts. In human cells, the well-documented alternative isoform has a 128 base pair insertion between positions 139 and 140 of the cDNA, resulting in three in-frame stop codons. An alternative translation start site in this insertion was proposed that would result in a REV3L protein with 78 fewer amino acids at the N-terminus (24). However, when it was experimentally tested whether N-terminal fragments of both isoforms could be expressed under their endogenous UTRs, only the reference isoform, and not the alternate isoform, was able to produce protein in vitro and in cellular translation systems (25). The alternative non-functional transcript isoform may be one way by which protein levels of REV3L are kept low in cells (25). Another means of limiting pol ζ levels may be proteolytic cleavage. A TASP1 cleavage site in REV3L is predicted by the eukaryotic linear motif resource (26) (Figure 2C). This would account for the prominent N-terminal proteolytic cleavage product of 60–70 kDa that is observed after REV3L overexpression (see Supplementary Figure S1 of (13)).

### Pol ζ disruption leads to mitochondrial deficits

Mitochondrial function depends on the coordinated expression of many nuclear genes. Consequently, mitochondria are a large and sensitive target and they provide a readout for nuclear genome stability. It is known that disruption of many nuclear DNA repair and metabolism genes can lead to mitochondrial dysfunction, and this appears to be true for pol ζ disruption as well (27).

It is not clear, however, whether active pol ζ is physically present in mitochondria or is only resident in the nucleus. It has been proposed that Rev3p contains a mitochondrial localization signal, but the reference mammalian *REV3L* sequence does not contain a functional mitochondrial localization signal (27,28). A suggested mitochondrial localization signal was identified in the predicted product of the shorter alternative mRNA isoform of mammalian REV3L (27). Ectopic expression of a peptide containing the N-terminal sequence of the alternate isoform drove the peptide to mitochondria (27), but there is no convincing evidence to show that this mRNA isoform produces REV3L (commercial REV3L antibodies are not able to detect REV3L in cells). There is currently no strong evidence for the presence of the other mammalian pol ζ subunits (or pol ζ function) within mitochondria.

### Pol ζ as a major extender enzyme past DNA lesions

**Figure 3.**
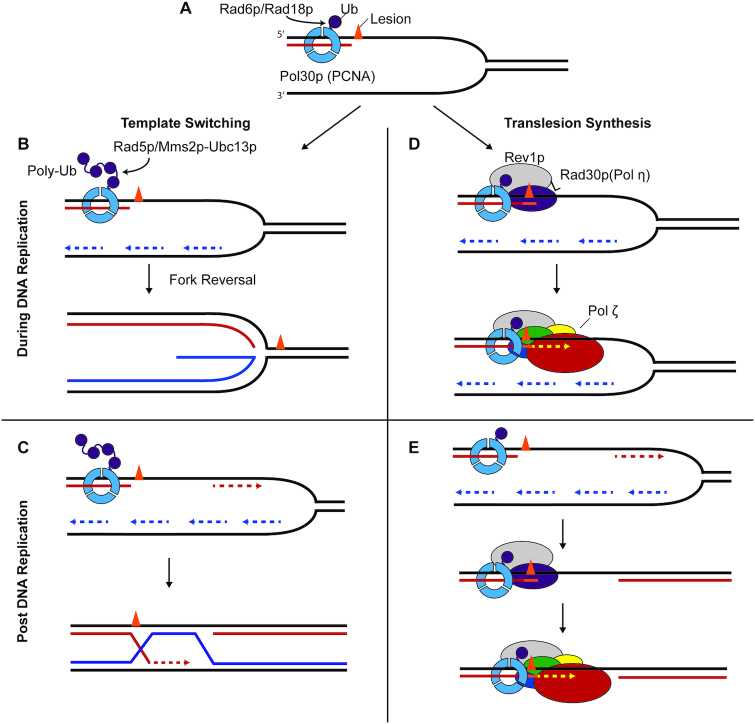
Models for lesion bypass. (**A**) When a replication fork stalls at a lesion, PCNA becomes monoubiquitinated by Rad6p/Rad18p at K164. This is the initial step in both lesion bypass pathways, translesion synthesis (TLS) and template-switching. For both pathways models have been proposed for bypass occurring at the replication fork (co-replication) or after synthesis has reprimed downstream of the lesion (post-replication). Here, bypass is depicted on the leading strand. (**B**, **C**) Template switching is instigated by polyubiquitination of K164, by Mms2p/Ubc13p/Rad5p. This pathway involves switching to the undamaged sister strand as a template for synthesis. This pathway may occur at the fork – one mechanism would be fork reversal as depicted here – or template switching may occur after the replication fork has moved on. (**D**, **E**) Rev1p binds monoubiquitinated Pol30p/PCNA (Ub-PCNA) facilitated by interactions through its BRCT, PAD, and UBM domains. A TLS pol such as pol η is recruited through interactions with Rev1 and Ub-PCNA. After recruitment, pol η (or another TLS pol) can insert a nucleotide opposite the lesion. Next pol ζ is recruited to Ub-PCNA by its interaction with Rev1p, and continues extension past the site of the lesion. In a co-replication model pol ζ would hand synthesis off to a replication polymerase. In the post-replication or ‘gap filling’ model, pol ζ may fill in a gap either on its own or might hand off to replication polymerases to finish synthesis in the gap.

Damage removal is usually the first line of defense against UV radiation or chemical adducts in DNA. For example, base excision repair (BER) removes abasic sites, and nucleotide excision repair removes UV radiation-induced pyrimidine dimers. If lesions are not removed by the time of DNA replication, they can block replication fork progression. In order for replication to reach completion the lesion must be bypassed. When replicative DNA polymerases stall at a lesion, there are two well-characterized routes of lesion bypass (Figure 3). In template switching, the lesion is circumvented completely by synthesis using the undamaged sister strand. In TLS, the lesion is directly bypassed by specialized DNA polymerases.

The route to either template switching or TLS is directed by posttranslational modifications to proliferating cell nuclear antigen (Pol30p/PCNA) (29–31). The homotrimeric Pol30p/PCNA forms a sliding clamp around the DNA, serving as an anchor for DNA polymerases. When a replication fork stalls at a lesion, Pol30p/PCNA becomes monoubiquitinated at K164 and promotes TLS. Alternatively, Pol30p can be polyubiquitinated at K164 (in yeast, by Mms2p/Ubc13p/Rad5p complex), and this shunts bypass to the template switching pathway.

TLS DNA polymerases and replicative DNA polymerases have contrasting biochemical properties that serve their respective functions. The high fidelity of replicative DNA polymerases is boosted by a functional exonuclease domain that corrects most errors. In contrast, TLS polymerases generally have low fidelity and lack a functional exonuclease domain. In Y-family DNA polymerases, this low fidelity has been attributed to a more spacious active site, which allows TLS DNA polymerases to incorporate bases across from lesions that cannot be accommodated by the active sites of replication polymerases (32). Another distinction between TLS polymerases and replicative polymerases is processivity, the amount of DNA synthesized in a single event before dissociation. TLS polymerases are less processive than replication polymerases. For example, Y-family TLS pol η, even when stimulated by the physiologically relevant monoubiquitinated PCNA, has processivity dramatically lower than pol δ (33). Low processivity may be selectively advantageous for organisms, preventing error prone polymerases from uninterrupted synthesis of large swaths of DNA which could leave genomes riddled with errors. Given the introduction of errors that occur with TLS, it makes sense that this is a tightly regulated process.

A large part of this regulation centers on monoubiquitinated Pol30p/PCNA, which serves as a docking site for key TLS players. For instance, the master TLS regulator Rev1p/REV1, binds monoubiquitinated Pol30p/PCNA facilitated by interactions through its BRCT, PAD and UBM domains (34,35). Rev1p/REV1 interacts with pol ζ via Rev7p/REV7 and Pol32p/POLD3 as described earlier (12,18–23). Rev1p/REV1 also interacts with Y-family TLS polymerases (36–38). This structural function of Rev1p/REV1, mediating key protein–protein interactions, is widely appreciated as essential to functional TLS (29–31,39,40). As discussed later, in certain scenarios Rev1p also contributes to TLS through its catalytic activity as a deoxycytidyl transferase (41).

Two models have been proposed for the timing of TLS: it may occur at the replication fork or after replication has continued through repriming past the lesion (Figure 3). These models are not mutually exclusive, and there is indirect evidence for both outcomes (42). In both models, monoubiquitination of Pol30p/PCNA instigates TLS, followed by association of Rev1p/REV1 and recruitment of a TLS polymerase.

Pol ζ has been implicated in direct bypass of several types of lesions as thoroughly reviewed previously (43). Correspondingly, cells with defective pol ζ are sensitive to an array of DNA damaging agents such as UV radiation, cisplatin and aflatoxin, which result in distorting or ‘bulky’ adducts on DNA (13,44,45). However, depletion of *Rev3l* in human cells does not alter survival or mutagenesis after BPDE treatment, another chemical that induces bulky adducts (46). Intriguingly, REV1, REV3 and REV7 were recruited to sites of DNA-protein crosslinks, implying that pol ζ may serve some function at these lesions (47). Additionally, pol ζ has been linked to interstrand crosslink repair (48,49), although biochemically Y-family polymerases appear to be intrinsically more efficient at bypassing interstrand links than pol ζ (50). Thus, pol ζ function at interstrand crosslinks have yet to be fully resolved.

On synthetic templates, Pol ζ can mediate insertion opposite some DNA adducts, as well as subsequent extension. Sometimes lesion bypass may be coordinated by two specialized polymerases. Pol ζ may play a role during lesion bypass by extending the primer-terminus after a nucleotide has been incorporated opposite a lesion by another TLS polymerase (51). For example, pol η and pol ζ have complementary biochemical activities that imply they are cooperating partners for lesion bypass. *In vitro*, pol η efficiently inserts a nucleotide opposite a 1,2 (dGpG) cisplatin intrastrand crosslink, while pol ζ cannot (7). In contrast, pol η starts to falter in extension past a lesion site, while pol ζ can efficiently extend past the lesion (7).

This introduces several important mechanistic questions. Does pol ζ continue significant synthesis after extending from a lesion? If the lesion is bypassed during DNA replication, when does pol ζ hand the reins back to the higher fidelity replicative polymerases? If lesion bypass occurs in a gap left after replication, can pol ζ fill the gap on its own?

### Extensive DNA synthesis beyond a lesion by pol ζ

There is evidence that pol ζ is capable of extensive synthesis downstream of UV-induced lesions in yeast. In yeast, Pol ζ-mediated synthesis can be monitored in vivo by mapping *REV3*-dependent mutations. When a plasmid containing a single tetrahydrofuran (a stable AP site mimic) is introduced into BER-deficient yeast cells, sequencing reveals mutations up to 200 bp away from the site of the lesion. These mutations depend on *REV3* function, indicating that pol ζ performs long range synthesis past the site of a lesion (52). In order to study *REV3*-dependent synthesis past the site of a UV radiation-induced photoproduct, reversion of an inactivating point mutation in the *URA3* gene was used. A TC sequence at the reversion site is a target for photoproduct formation by UV radiation. Following UV irradiation, *REV3*-dependent mutations were detected up to 1 kb away from the UV-induced target site. Single-stranded gaps in this size range are observed after UV irradiation in NER-deficient yeast, implying that pol ζ may be responsible for synthesis in a large portion of such gaps (52,53). Synthesis could occur during genome replication or afterwards. In either case, these results suggest that mutagenic DNA synthesis by pol ζ extends significantly beyond a site of UV radiation induced damage.

### Unique biochemical characteristics of pol ζ

Pol ζ is the only specialized DNA polymerase in eukaryotes that is a member of the B-family; the other specialized DNA polymerases belong to the A, Y, X and AEP families. The error rate of pol ζ is intermediate between replication and TLS DNA polymerases. It is 10 to 100-fold less accurate than its fellow B-family members in the DNA replication apparatus, but 5 to 30-fold more accurate than Y family TLS polymerases (54). However, much of this analysis was conducted using Rev3p and Rev3p-Rev7p, before pol ζ was discovered to include Pol31p and Pol32p. The relative processivity of pol ζ has yet to be evaluated systematically. Given *in vivo* evidence for pol ζ long-range synthesis, it would be useful to measure its processivity in a single round of DNA synthesis in comparison to Y family polymerases. These unique biochemical features of pol ζ may be relevant not only to its function in lesion bypass, but to more recently revealed pol ζ functions.

### Pol ζ synthesis associated with DNA replication stress

In yeast, pol ζ is responsible for a substantial portion (half or more) of ‘spontaneous’ mutations in cells that are not challenged with external DNA damaging agents (55–57). This implies that cells use pol ζ to copy DNA containing endogenous lesions (such as AP sites) and in response to other stresses. One source of DNA replication stress can be induced through depletion of dNTP pools by incubation with hydroxyurea (HU). Such exposure to HU induces point mutations in yeast, which are largely dependent on *REV3* (58). Additionally, DNA synthesis by pol ζ was not dramatically altered by changes in dNTP levels *in vitro* or *in vivo*, unlike other DNA polymerases (59). Together this data is consistent with a model whereby pol ζ synthesis plays a role in dealing with replication stress, including circumstances where dNTP levels are low enough to hinder chromosomal replication. However, there are multiple pathways for cells to deal with nucleotide depletion since deletion of *REV3* in yeast does not confer enhanced sensitivity to HU (60). Interestingly, knockdown of ribonucleotide reductase M1 (which should result in lower dNTP pools) decreased the viability of *REV3L*-defective human cells more than *REV3L*-proficient controls (61). More research may help clarify the roles mammalian pol ζ may play when cells are under replication stress.

Pol ζ-dependent mutagenesis in yeast also occurs following other types of replication stress. Mutations in replicative polymerases can cripple the replisome and result in elevated mutagenesis. This has been called DRIM or defective-replisome-induced-mutagenesis. Importantly, the vast majority of this mutagenesis is dependent on *REV3* (58,62). Furthermore, the ability of Rev3p to interact with Pol31p–Pol32p is essential for DRIM, implying that pol ζ functions as a four-subunit complex in DRIM (63). Additionally, *REV3*-dependent mutations in DRIM increase when the template switching pathway is inhibited by deletion of *MMS2*. This illustrates the importance of PCNA directing repair choice to either pol ζ synthesis or the ‘error-free’ template switching when the replisome runs into trouble. Together this work demonstrates that there are multiple routes for cells to overcome various sources of replication stress, and that pol ζ synthesis is an important but mutagenic route.

**Figure 4.**
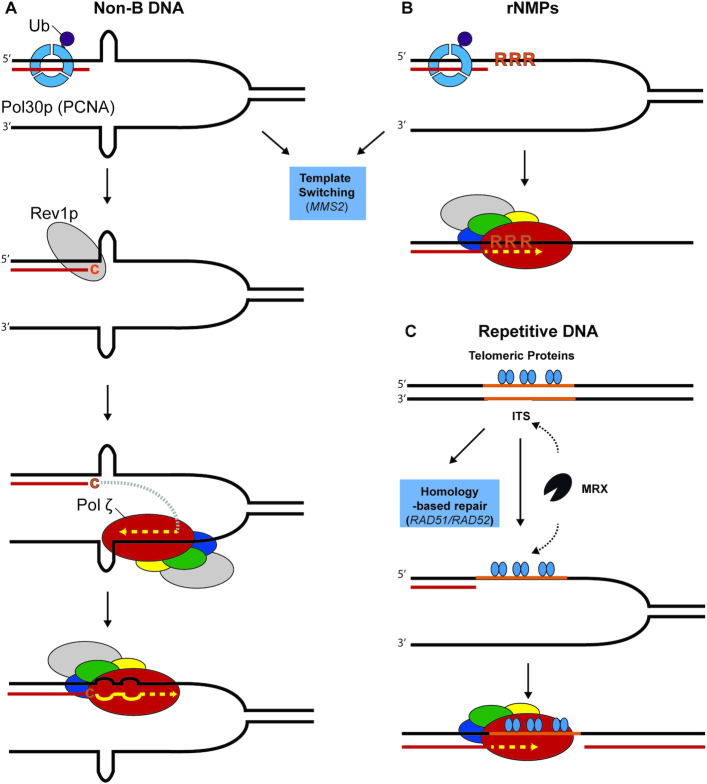
Models for *REV3*-dependent synthesis at difficult-to-replicate regions in the genome. (**A**) Model, after Northam *et al.*, for the role of pol ζ and Rev1p synthesizing non-B DNA (66). Hairpin structures in DNA can stall replicative polymerases. Ubiquitinated PCNA then recruits Rev1p. Rev1p first acts enzymatically and inserts a dCTP near the base of the hairpin, after which it serves to recruit pol ζ. Pol ζ then initiates synthesis on the opposing strand followed by repriming near the original site of the hairpin, copying from this mismatched sequence. Alternatively, hairpin structures can be bypassed by template switching. (**B**) Model for pol ζ bypass of incorporated ribonucleotides proposed by Lazzaro *et al.* (75). Incorporated ribonucleotides can be repaired by ribonucleotide excision repair or a Top1-dependent pathway. When incorporated ribonucleotides remain in the DNA during replication they have the potential to stall a replication fork. In yeast, pol ζ may bypass incorporated ribonucleotides. (**C**) Model for pol ζ replicating interstitial telomeric sequences (ITS), after Moore *et al.* (76). Telomeric proteins, at (ITS), may result in stalling of the replication fork. *REV3* is proposed to fill a gap caused by cleavage of the fork by the MRX endonuclease complex (Mre11p/Rad50p/Xrs2p) followed by end resection. Alternatively, cells can deal with ITS through homology dependent repair mechanisms (dependent on *RAD51* or *RAD52*).

A further source of replication stress arises when the replisome encounters DNA sequences that are challenging to replicate, such as sequences forming secondary structures (64,65) (Figure 4A). Spontaneous and DRIM pol ζ-dependent mutations often occur at predicted sites of hairpin formation in vivo (66). These same hairpin structures stall pol δ *in vitro*. This highlights that introduction of *REV3*-dependent mutations can be sequence-specific, and could explain variability in the *REV3*-dependent mutation rate when different regions are monitored. DRIM is also dependent on the deoxycytidyl transferase Rev1p. Mutations in response to replication perturbations were reduced in a *REV1*-deletion strain as well as in a strain with a deletion in the *REV1* BRCT domain mediating protein-protein interactions. This is consistent with Rev1p having both a structural and enzymatic function in bypass of DNA structures that are difficult to replicate (66).

### Bypass of incorporated ribonucleotides

Another major source of replication stress arises from ribonucleotides (rNMPs) erroneously incorporated into the genome during replication (67). Multiple pathways exist to repair and bypass rNMPs, which likely represent the most common error made during replication (68–72). One study has suggested that pol ζ bypass may be one of these pathways.

Most incorporated ribonucleotides are removed from DNA by ribonucleotide excision repair. This process is initiated by RNase H2 (and perhaps RNase H1). An alternative repair mechanism exists that is dependent on topoisomerase I (73,74). However, if unrepaired rNMPs are present during replication they may be bypassed. Incorporated ribonucleotides can also be bypassed by the *MMS2-*dependent template switching pathway discussed above. Yeast cells lacking RNase H1, RNase H2 and Mms2p are only mildly sensitive to HU, but co-deletion of pol ζ components in these cells dramatically sensitizes them to HU (75). This suggests that pol ζ provides one mechanism for cells to survive replication fork stalling induced by collision with rNMPs (Figure 4B).

Providing biochemical support for this model, two-subunit pol ζ (Rev3p-Rev7p) is dramatically more efficient than pol δ at bypassing four tandem rNMPs (75). It will be intriguing to see if the four-subunit pol ζ (Rev3p-Rev7p-Pol31p-Pol32p) can bypass rNMPs even more efficiently than Rev3p-Rev7p alone. In addition, it will be important to determine if this function is conserved for mammalian pol ζ.

### Pol ζ and repetitive DNA sequences

Highly repetitive sequences are another impediment to replicative polymerases. An important example are telomere sequence repeats that are found embedded in chromosomes outside of the telomeres, called interstitial telomeric sequences (ITS). When an ITS was placed within an intron of *URA3*, an increased rate of point mutations and terminal sequence inversions was observed in adjacent gene segments (76). The point mutation rate was reduced 10-fold in a *rev3* deletion strain (76). These *REV3*-dependent mutations appeared not to result from a repair of a double strand break, since the mutations were not accompanied by indels in the ITS. The mutations were also dependent on Mre11p, a component of a nuclease that acts on stalled replication forks and facilitate their degradation. This led to the model that *MRE11*-dependent gaps are formed at ITS sequences and are then filled in by pol ζ (Figure 4C). Mutations adjacent to the ITS increased upon disabling homology-based repair by deletion of *RAD51* or *RAD52*, but not after disabling the template switching pathway by deletion of *MMS2*. This suggested that homology-based repair pathways are an alternative repair mechanism at ITS sequences, while canonical template switching is not.

*REV3*-dependent mutations in the neighborhood of other types of repetitive sequences have been observed. Mutations in a *URA3* reporter gene were observed as far as 8 kb past an Alu inverted sequence quasi-palindrome; these mutations were largely *REV3*-dependent (77). Mutations also occur in the neighbourhood of trinucleotide repeats (78,79). *REV3*-dependence of mutations in the chromosome adjacent to trinucleotide repeats was most apparent in strains with replisomes carrying crippling *pol3* or *pol2* variants (77,80). While it was demonstrated that double strand breaks do form at these repeats, it is not known whether the pol ζ mutations are dependent on the formation of a break.

**Figure 5.**
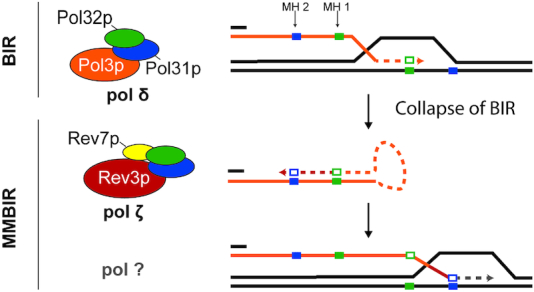
Model for the role of pol ζ in microhomology-mediated break-induced replication. In break-induced replication (BIR) a single-ended DNA break is repaired by extensive synthesis on the sister by pol δ. If the replication fork in BIR collapses, an alternative repair pathway called microhomology-mediated BIR (MMBIR) can be used. A proposed model for MMBIR involves looping of a strand, annealing to itself at a short region of homology (microhomology, MH), with pol ζ carrying out DNA synthesis from this MH-paired primer. The extended strand re-associates with the sister strand, annealing at a microhomology created during synthesis, and continues synthesizes using the sister template. This model was proposed by Sakofsky *et al.* (84).

It has been known for two decades that *REV3*-dependent mutagenesis occurs in reporter genes located in the neighborhood of a double strand break induced by the HO endonuclease (81,82). Nevertheless, *REV3* is not essential for the repair of the break, leaving the question of the function of pol ζ synthesis around double strand breaks (81,83). Pol ζ is necessary for a specific type of single-ended DNA double strand break processing, termed microhomology-mediated break induced replication (MMBIR, Figure 5) (84). Single-ended double strand breaks can arise from collapsed replication forks or degradation of telomeres. Cells can repair the break through extensive synthesis, involving pol δ, called break-induced replication (BIR, Figure 5). BIR uses the homolog as a template and a primer with a substantial stretch of homology. In yeast BIR has been monitored by the induction of a single ended break at the end of a single chromosome III by HO endonuclease, and tracked with auxotrophic reporters. Deletion of the *PIF1* helicase gene disables extensive synthesis, resulting in collapse of BIR and the appearance of complex mutations near the site of strand invasion (84). These complex mutations can be explained by synthesis arising from alternative templating at microhomologies. These mutations, in *pif1Δ* yeast, are thought to arise from MMBIR, and such mutations were absent in strains with deletion of *REV3* (84). Point mutations downstream of the single-ended break repaired by MMBIR have not been measured. Such experiments could provide insight into the extent of pol ζ synthesis and its potential function during MMBIR.

An intriguing parallel arises from recent work in human cells, where restart of stalled replication forks in *BRCA2*-deficient cells was dependent on the MUS81 endonuclease and POLD3 (85). A model proposed from this work was that reversed forks (Figure 3B) were being cleaved by MUS81, forming a single-ended DNA break that was repaired with POLD3-dependent synthesis. Whether this repair is mediated by pol δ, pol ζ, or combination of the two remains unresolved.

### Growth defects in pol ζ disrupted mammalian cells

Yeast genetics resulted in the discovery of *REV3* over four decades ago, and yeast studies are still at the forefront of unravelling the multifaceted functions of pol ζ as a specialized DNA polymerase. *REV3* mutants of *S. cerevisiae* are viable, without an enhanced formation of gross chromosomal rearrangements (86). In marked contrast to yeast, inactivation of Pol ζ has severe consequences for mammalian cells without exogenous sources of stress. Clarifying this essential function in mammalian systems is paramount to understanding the relevance of pol ζ to humans and cancer.

Without pol ζ function, mammalian genomes accumulate DNA breaks in every cell cycle, with ensuing chromosome breaks, gaps, rearrangements, and formation of micronuclei. In normal cells, this DNA damage impairs viability by initiating gene expression programs that lead to cell death or senescence. For example, primary mouse embryonic fibroblasts (MEFs) die or senesce within two cell divisions after disruption of *Rev3l*. This excessive cell death and premature senescence in primary MEFs can be prevented by immortalizing cells with T-antigen before disruption of *Rev3l*. Although cells can proliferate because immortalization blunts the implementation of cell death pathways, the *Rev3l* defect leads to a 10-fold increase in the frequency of DNA double-strand breaks and chromosome rearrangements (6,87). Similarly, some human cancer cell lines such as NALM-6 can propagate after inactivation of *REV3L*, but with increased sensitivity to many DNA damaging agents (88). A *REV3l*^−/−^ mutant was also derived from TK6, a p53^+/+^ EBV-immortalized lymphoblastoid cell line (89). Presumably such cell lines are also compromised in checkpoints or cell death pathways. An interesting parallel arises from studies of yeast strains with defective telomere maintenance. Such strains with a *rev3* deletion cannot proliferate and undergo senescence (90). One proposal is that short inverted repeat-induced synthesis is used to maintain chromosome ends, taking advantage of palindrome sequences (90). This process, related to MMBIR, may require DNA synthesis mediated by pol ζ.

Mouse embryos with a homozygous disruption of *Rev3l* do not develop further than mid-gestation. Some strains lose viability even earlier, and so several investigations have turned to conditional disruption models. A broad inactivation of *Rev3l* in hematopoietic progenitors does not give rise to propagating B or T cells in adult mice (91). Disruption is better tolerated in mature resting B cells expressing CD19 or CD21 (92,93).

Adult mice are able to survive after ablation of *Rev3l* in epithelial keratinocytes (using Cre recombinase driven by a keratin-5 promoter) (87). However, these animals display major skin abnormalities, including reduced epidermal cellularity, irregular hair cycling, and gradual hair loss (87). Keratinocytes from the mice exhibit highly enhanced baseline levels of DNA damage stress and do not survive if explanted into cell culture. Remarkably, these mice are the most sensitive to UV radiation of any known single mutant mouse model, much more sensitive than those with defects in nucleotide excision repair (87).

The greatly enhanced UV radiation sensitivity of mice with *Rev3l*-defective keratinocytes arises from two sources. First, the cells are defective in TLS of UV radiation-induced DNA damage. Second, the pol ζ defect impairs the proliferation of normal keratinocytes as the skin attempts to recover from UV-irradiation. To verify that pol ζ is necessary for continued proliferation of primary cells in the mouse, even in the absence of exogenous DNA damage, wound healing was examined. This process depends on rapid proliferation of cells to regenerate the wounded area. *Rev3l*-defective epidermis was found to have a proliferative defect in wound healing (94). Pronounced epidermal skin pigmentation occurred following UV irradiation or wound healing in *Rev3l*-defective adult mice (87,94). This is striking because, unlike humans, adult mice do not normally have melanocytes resident in the basal epidermis. The explanation is related to the DNA damage-dependent stress response developed by *Rev3l*-defective keratinocytes as they attempt to proliferate. Stress signals from the keratinocytes then induce p53-dependent migration of melanocytes to the epidermis (94). In keratinocytes, p53 directly upregulates the transcription of genes that stimulate production of eumelanin and increase pigmentation (95–98).

### Pol ζ as a tumor suppressor

One consequence of the genome protective function of pol ζ is that *Rev3l* is a suppressor of spontaneous tumor formation. There are several examples. In *Tp53*-defective mice, a mosaic deletion of *Rev3l* in the lymphocyte lineage caused an increased incidence and reduced latency of lymphomas (91). Conditional partial disruption of *Rev3l* in the mammary gland enhances the incidence of mammary gland tumorigenesis (91). About 90% of mice with disruption of *Rev3l* in keratin-5 expressing cells acquired squamous cell carcinomas after 1 year (87). It is likely that the increased frequency of tumors is due to oncogenic chromosome rearrangements in *Rev3l*-defective tissues. For example, cancers arising from *Tp53*-defective and *BRCA1* defective mouse epithelia are driven by amplifications or translocations that result in elevated oncoprotein expression or oncoprotein­containing fusions (99).

### The polymerase activity of pol ζ is essential for limiting genome damage

One question is whether the essential functions of REV3L depend directly on the DNA polymerase, or whether only the structural presence of REV3L is needed. A catalytically deficient REV3L might still interact with its protein partners and other DNA substrates, allowing viability of cells and mice. To determine whether ablation of the catalytic activity of pol ζ is responsible for the severe consequences in pol ζ mutant mouse cells, mice were engineered with specific inactivation of the catalytic activity. Conserved active site aspartate residues necessary for activity (D2773, D2775) were changed to alanine, in two independent studies. In both cases, no viable homozygous mice were produced, and the corresponding embryos died early in embryogenesis (6,100). To investigate cell-autonomous consequences of the specific polymerase alteration, primary MEFs were derived that carried one null *Rev3l* allele, and one active site mutant allele (6). The growth defects, DNA break formation and cisplatin sensitivity of these cells were similar to cells harboring two null alleles (101). These results showed that the DNA polymerase activity of REV3L is essential for the genome protective functions.

### Different consequences of *REV7* and *REV3L* knockouts in mammals.

As we noted earlier, REV7 (encoded by *MAD2L2*) functions as a component of pol ζ. A dimer of REV7 binds to two adjacent sites in REV3L by grasping a peptide of REV3L with a ‘safety-belt’ loop (13,14). REV7 serves as a bridge between REV3L and the REV1 protein (Figure 1B). The REV7 protein is, however, at least an order of magnitude more abundant than REV3L (13) and has additional functions and numerous protein partners. These include chromatin-associated proteins and post-translational modification proteins (102–108). A further function of REV7 has been established as a DNA resection inhibitor in the ‘Shieldin’ complex (109,110). In view of these many functions, it is fascinating that complete ablation does not always seem to block viability of mice. *Rev7* disruption in mice (using embryonic stem cells derived from 129 strains) gave homozygous progeny in a sub-mendelian ratio, with a smaller overall size and pronounced defects in primary germ cells (105,111). Germline deletion of *Rev7* in C57BL/6 embryonic stem cells resulted in embryonic lethality (112). Specific deletion of Rev7 from the B cell lineage using *Mb1-Cre* yielded normally functioning B cells, unlike the severe defects in *Rev3l*-defective B cells (112). These considerations suggest that *Rev3l* has as yet unappreciated functions that are independent of Rev7, and perhaps independent of TLS. Studies that elucidate the broad consequences of *Rev3l* deletion, such as profiling the changes in the transcriptome and proteome, may help uncover novel functions of pol ζ.

### Cancer and disease-associated mutations in *REV3L*

*REV3L* is a large gene and therefore many *REV3L* mutations are found in the genomes of human cancers. *REV3L* is mutated in more than 10% of the NCI-60 cancer cell line set (113). Analysis via cBioportal of cancers with both mutation and copy number data currently shows that *REV3L* is altered in ∼2% of cancers and that ∼18% of these mutations occur with loss of heterozygosity (114,115). Heterozygous mutations in the human *REV3L* gene have also been suggested to be associated with an inherited neurological disorder, Möbius syndrome (116). It will be interesting to investigate whether the association can be confirmed in further studies of mouse models and the human disorder.

### Inhibition of pol ζ as a potential therapeutic target

Genomic instability fuels cancer development and also provides a reservoir of genomic variations that facilitate resistance to therapies. Tumor cells often have altered DNA repair pathways, resulting in genomic instability, one of the defining hallmarks of cancer (117). However, this can lead tumor cells to rely more heavily on remaining intact repair pathways to prevent genomic damage from reaching levels that lead to unescapable cell death. As a result, targeted therapies have emerged that exploit specific DNA repair deficiencies in tumor cells, the most prominent being the rise of PARP inhibitors in the clinic (118).

A potentially exciting prospect for cancer therapy is to inhibit the function of TLS in tumor cells to block a remaining route of survival of cancer cells challenged with DNA damaging chemotherapeutics. Depleting pol ζ function should increase sensitivity of a tumor to some DNA damaging drugs. Concomitantly, reduction of the TLS function would be expected to reduce drug-induced base substitution and frameshift mutagenesis. This could advantageously reduce the pool of drug-induced mutated tumor cells, which could slow down recurrence of a tumor.

This strategy has been explored in mouse tumor models. Mouse lung adenocarcinoma cells were transplanted into syngeneic mice, and the animals exposed to cisplatin (119). Cisplatin toxicity was higher in *shRev3l*-depleted cells, and mice receiving tumors harboring *shRev3l* had a longer survival time following cisplatin treatment (22.5 days) compared to control tumors with normal Rev3l levels (11 days). The mutation frequency of cells expressing *shRev3l* was ∼20% of normal following cisplatin treatment. Other experiments used a model of Burkitt lymphoma, the *Eμ-myc* mouse (120). Pure populations of control and *Rev3l*-deficient lymphoma cells were introduced into recipient mice until palpable tumors formed (∼2 weeks). Following treatment with a single 10 mg/kg dose of cisplatin, *Rev3l*-deficient tumors exhibited a more rapid reduction in size than controls, with tumor regression occurring within 24 h.

Other experiments focused on REV1 (120). To examine whether *Rev1* deficiency similarly could delay the development of chemoresistant tumors in vivo, control and *Rev1*-deficient *Eμ-myc* lymphoma cells were injected into syngeneic recipient mice. After tumors formed, mice were treated with cyclophosphamide. To examine the role of *Rev1* in the evolution of chemoresistance, tumors were harvested from individual mice at relapse and reinjected into mice for a second round of therapy. Even after three cycles of engraftment, treatment and regrowth, three of four *Rev1*-deficient tumors retained a pronounced sensitivity to CTX treatment, whereas all control recipients succumbed to their tumors. These data suggest that TLS inhibition may have dual anticancer effects, sensitizing tumors to therapy as well as preventing the emergence of chemoresistance.

Because pol ζ inactivation is toxic to normal cells, a major practical challenge is the development and delivery of a pol ζ inhibitor to tumor cells. In one approach, a nanoparticle was used to deliver a cisplatin prodrug and REV1/ REV3L-specific siRNAs simultaneously. Administering such nanoparticles to a prostate cancer cell mouse model gave a synergistic inhibition of tumor growth (121).

Another approach may be to target small-molecule inhibitors to the pol ζ complex (122). A further strategy is to inhibit specific interactions between proteins in the extended pol ζ complex. Protein-protein interactions between the C-terminal domain of REV1 and the REV1-interacting region of other TLS DNA polymerases play an essential role in TLS. A fluorescence polarization-based assay was used in a pilot screen for small molecule inhibitors of this PPI (123). Small molecule scaffolds that disrupt this interaction were identified. Survival and mutagenesis assays in mammalian cells exposed to cisplatin or UV radiation indicated that these compounds inhibit mutagenic REV1/pol ζ-dependent TLS in cells (123).

**Figure 6.**
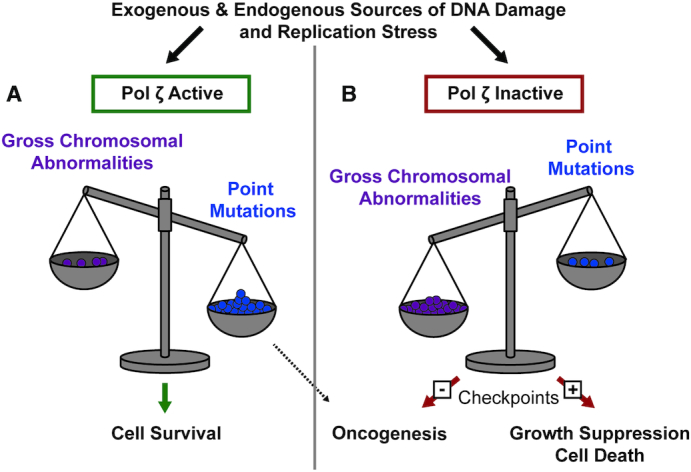
Pol ζ operates as a tumor suppressor by preserving chromosomal stability at the cost of point mutations in mammals. (**A**) In cells with proficient pol ζ, endogenous and exogenous sources of stress or damage require pol ζ synthesis to prevent gross chromosomal abnormalities, but at the cost of introducing point mutations. (**B**) Without pol ζ, cells may accrue fewer point mutations but also acquire increased chromosomal abnormalities when challenged with exogenous or endogenous sources of damage or stress. These chromosomal abnormalities lead to growth suppression and cell death in cells with functional checkpoints. Cells without functional checkpoints may be able to survive and the chromosomal abnormalities can drive oncogenesis.

There are many other protein–protein interactions within the pol ζ complex (20) (Figure 1). Some of these interactions are candidates for small-molecule targeting. Specific inhibitors of the catalytic activity of REV3L could also inhibit pol ζ function (6,100). Recently an inhibitor that prevents the REV7-REV1 interaction through promoting dimerization of REV1 was identified (39). Importantly, when combined with cisplatin, this inhibitor reduced tumor size and increased survival in a mouse xenograft model. An important caveat is that disruption of *Rev3l* function causes cells to be susceptible to increased chromosome breaks, gaps, and oncogenic chromosome rearrangements (Figure 6). Crippling of REV1 function (in a nucleotide excision repair-defective background) enhances UV radiation-induced carcinogenesis by elevating inflammatory hyperplasia (124). Therefore, pol ζ inhibition could sometimes contribute to generating drug-resistant variants in recurrent cancer, and such possibilities will have to be monitored carefully.

## CONCLUSION

Pol ζ is a specialized DNA polymerase that functions in canonical translesion synthesis but also has broader roles in unchallenged replicating cells. Uncovering the roles pol ζ plays in preserving genomic stability in mammalian cells could have broad reaching implications in understanding carcinogenesis and in developing combination therapies for some cancers.
